# Use of SSR-Tools for clone certification in Uruguayan *Eucalyptus grandis* and *Eucalyptus dunnii* breeding programs

**DOI:** 10.1186/1753-6561-5-S7-P58

**Published:** 2011-09-13

**Authors:** Diego Torres-Dini, Zohra Bennadji, Milton Cabrera, Carmelo Centurion, Fernando Resquin, Gustavo Balmelli

**Affiliations:** 1 Forestry Biotechnology Laboratory, National Institute for Agriculture Research. INIA Tacuarembó, Uruguay; 2 Forestry Breeding Program, INIA Tacuarembo, Uruguay; 3 Forestal Oriental, UPM, Paysandú, Uruguay

## Background

Over the last two decades, commercial plantations in Uruguay have increased exponentially, particularly those destined for pulp and paper industry. Although the about 700,000 ha of commercial plantations of Eucalyptus are a continuous source for the selection of elite genotypes, there is no national registration system for Eucalyptus clones in this country. The traditional procedure of clonal identification accepted by the UPOV (International Union for the Protection of New Varieties of Plants) involves the evaluation of morphological descriptors such as leaf shape, bark texture, fruit shape, etc. [1]. The assessment of these characters varies from one observer to another, potentially leading to ambiguous results [2,3]. This restriction has led countries such as Brazil to include the use of microsatellite markers as additional descriptors, in their legislation. The hypervariability and single inheritance of microsatellite markers provide a powerful clonal characterization system through fingerprinting. The identification of elite clones is nowadays one of the most widely used applications of molecular markers. This could generate a labeling system to follow the material traceability in companies with large-scale production of clonal nurseries. Proper identification makes new clones releasing easier and improves the management of seed orchards and controlled pollination breeding programs [4-6]. The aim of this study was to characterise 24 elite clones of several breeding programs in Uruguay, by the use of microsatellites. This was achieved by verifying the potential for discrimination of these molecular tools by assigning a specific molecular pattern of fingerprinting to each tested clone.

## Methodology

Plant material was collected from 24 elite clones of Eucalyptus, provided by INIA Uruguay and Forestal Oriental. They were subdivided in 14 samples of E. grandis and 10 of E. dunnii. The tested clones were analyzed by PCR with 5 microsatellite loci using EMBRA 4, EMBRA 5, EMBRA 10, EMBRA 11 and EMBRA 16 markers under the same conditions as previously reported [7,8]. The molecular weight of the alleles was estimated with an automatic sequencer Macrogen Koerea [http://www.macrogen.com/eng/macrogen/macrogen_main.jsp] that employed Peak Scanner software V1.0 [https://products.appliedbiosystems.com]. The data obtained for each microsatellite was subjected to statistical assay to determinate the following parameters: the probability of identity (PI), the paternity exclusion probability (Pe), the heterozygosity (He) and the observed heterozygosity (Ho). This analysis was performed with the software IDENTITY 1.0 [9].

## Results and discussion

The generated data was sufficient to assign a specific profile for each tested clone. The five EMBRA markers amplified a total of 90 alleles and a maximum of 23 alleles for the EMBRA 11 marker. The minimum was found for the EMBRA 16 marker with 14 alleles. The remaining three markers showed 20, 18 and 15 alleles per locus.. The probability of identity (PI) was 5.43 x10-9 and the paternity exclusion (Pe) 0.999. The expected heterozygosity (He) for all loci ranged from 0.894 to 0.958 while the observed heterozygosity (Ho) showed values ranging from a minimum of 0.471 to a maximum of 0.936 for every loci. A likelihood value of complete identity of 5.43 x10-9, guarantees the individual identity of each clone, as the probability of two being equal by chance is 1 in 184 million. A precise molecular pattern for each of the 24 genotypes could be assigned and the information produced is sufficiently robust to ensure the molecular traceability for each of these materials at the different stages of nursery production and/or field trials. However, the five markers derived from genomic sequence containing dinucleotide repeats. These markers, even when making a great discrimination, have reduced precision of genotyping, necessary for comparative multilocus profiling across laboratories or even at different times in the same equipment [10]. Considering a National System of Clones Registration, it proves necessary to increase the number of markers employed by including tetra and pentanucleotide markers to strengthen the precision and reproducibility among different laboratories. In addition, special emphasis should be laid on their selection, focusing on those microsatellites with high interspecies transferability. The testing of different species of Eucalyptus with the same molecular methodology and standardized procedures, will contribute to the registration of elite material and the protection of intellectual property.

## Conclusions

The results show that using five microsatellite markers it is possible to discriminate among 24 clones and assigning a molecular fingerprint characteristic for each of them. The transferability of the five markers used is also verified, since they amplified both *E. grandis *and *E. dunnii*.
